# Out-migration and attrition of physicians and dentists before and after EU accession (2003 and 2011): the case of Hungary

**DOI:** 10.1007/s10198-016-0854-6

**Published:** 2016-12-02

**Authors:** Júlia Varga

**Affiliations:** Institute of Economics, Centre for Economic and Regional Studies, Hungarian Academy of Sciences, Budaorsi ut 45, Budapest, 1112 Hungary

**Keywords:** Medical doctors’ migration, Attrition, Competing risk model, EU enlargement, C41, C55, I10, J4, J40, J45, J60, J61

## Abstract

This paper employs a large-scale, individual-level, panel dataset to analyse the effect of EU accession on the probability of out-migration on the part of Hungarian physicians and dentists between 2003 and 2011. The study uses event history modelling and competing risk models. The results show that EU accession did not at the time affect the probability of out-migration while after the end of the transitional period of restrictions on the free movement of labour from the new EU member states to Austria and Germany, the probability of doctors’ migration increased considerably. Relative wages and peer pressure also exercise a significant role in the out-migration decisions of young medical doctors. We also find that more than half of those medical doctors who left the country during the observation period returned some time later. The data furthermore suggest a massive flow of doctors to domestic jobs outside the health care system.

## Introduction

The aim of this study is to analyse how European Union (EU) accession has affected the emigration of medical doctors. This is increasingly the object of keen attention in many European countries because it places additional pressure on an already constrained workforce [1–3]. Demand for medical doctors continues to exceed supply for reasons such as increased training costs, an aging population, the development of medical technology and growing degree of specialisation [4–6]. Furthermore, increasing pressure at work has resulted in medical doctors leaving clinical practice for less stressful types of jobs, e.g. medical advisor or medical sales representative [7]. The European Commission predicts that the EU will face a shortage of 230,000 physicians and a further shortage of 150,000 dentists, pharmacists and physiotherapists by 2020 if existing workforce problems are not addressed [8].

All EU member states are losing medical doctors, not only the Central and Eastern European new member states (EU-10^1^), but also the EU-15^2^ countries. However, unlike the EU-10 countries, many EU-15 countries are the destination of migration by health professionals from other nations [3, 9–13]. The share of foreign trained and foreign born doctors on average is lower in the EU10 than in EU15 countries, (in 2012–2014 it was 4.5 versus 13.4% and 6 versus 13.9%, respectively^3^) [14]. In Hungary the share of foreign trained doctors is relatively high compared to other EU10 countries. It was 7.6% in 2012–2014, but the share of foreign born doctors was as low as in other EU10 countries, at 2.1% in the same period [14]. As the EU-10 countries are currently not competitive with the old EU member states in terms of wages, working conditions and career prospects, EU accession amplified fears in the Central and Eastern European new member states that accession may facilitate the movement of physicians from these countries to other parts of the Union, and that out-migration could intensify shortages of doctors in these countries.

Several projects (MoHProf,^4^ PROMeTHEUS^5^) and studies [3, 9–13, 17–20] have examined the migration of medical doctors in Europe. These studies concluded that post-accession outflows from the Central and Eastern European new member states have been lower than expected, but they also found that more recent data show some increase in outward migration [21]. Nevertheless, most of these studies emphasise that there is a lack of reliable data on out-migration (e.g. in most countries, the only data source available to estimate outflows is ‘intention-to-leave’ data, that is, the number of applications for certificates of recognition of diplomas or survey data asking about medical doctors’ intention to work abroad. These data have limited reliability in relation to the movement trends of doctors because not everyone who applies for certificates or who is planning to leave their home country actually leaves. Also, individuals may apply more than once, leading to overestimates of actual flows. Furthermore, not all countries systematically request these certificates, and this may result in the underestimation of migration [2, 16, 17, 22, 23]). In addition to the difficulties of the measurement of outward migration, we know far less about how return migration has changed. The few studies that have investigated return migration used survey data to assess the magnitude of it [e.g. 24].

Our research explores a unique, large-scale, individual-level, panel data set that allows us to follow out-migration, attrition and other employment status changes of Hungarian medical doctors at the individual level on a monthly basis between 2003 and 2011. This study may supplement previous research as it analysed actual changes in out-migration and employment status of medical doctors. In addition, our sample also made the observation of the return migration of physicians during the observation period possible. The paper further provides estimations on how the probability of return migration has changed between 2003 and 2011.

## Data

The base data set is a large, merged, longitudinal dataset covering 50% of Hungary’s population aged 5–73 in 2003. The data contains information from registers of the Pension Directorate, the Tax Office, the Health Insurance Fund, the Public Employment Service and the Office of Education. From this source sample, a medical doctors’ subsample was created. All individuals were included in the medical doctors’ subsample whose occupation code was “medical doctor, general practitioner”, “medical doctor, specialist doctor” or “medical doctor, dentist” according to the Hungarian Occupational Classification system for at least 1 month between January 2003 and December 2011. Each datum in the sample is followed until December 2011 or exit from the social security system (for reasons of death or permanent emigration). The maximum number of observations for one individual is 108 months. The unit of observation is the monthly status of individuals.

Our data contain information month by month on the labour market status of the individual; whether the individual was working for the given month in Hungary; if so, what his/her occupation code was; also the question of whether the person was studying, in receipt of benefits such as old-age pension, disability benefit, or child care allowance and social security benefits, unemployment insurance benefit, or unemployment assistance. We also have data on demographics (date of birth, gender), the region of residence and the labour income of the individual. The income from employment contains any informal payments the individual doctor listed in their tax statement. The use of informal health payments or “thank-you money” to gain access to health care is widespread in Hungary, as in most Eastern European countries [e.g., 25, 26]. Although doctors have to declare informal payments in their tax statements, tax evasion is also widespread, and most tax statements include only a part of the real amount of such payments. We could not address the problem of this hidden income in this study.

We have data for 18,266 individuals. The number of working doctors varied between 9594 and 11,415 persons in the different observed months, and of these, 2933–3331 were general practitioners, 5462–6899 specialist doctors, and 1024–1469 dentists. (Summary statistics for the variables used in estimation are presented in Appendix 1 Table 7). There are some biases in the sample. First, as we do not have data on medical graduates who have never worked in the Hungarian health system, they are not included in the sample. Other data on medical graduates indicates high outward migration for graduates. For example, about 40% of those who finished their studies between 2007 and 2010 have not yet registered in the system [27]. As we could not include medical graduates in our analysis, our results give a lower bound estimate on the out-migration of Hungarian doctors. Secondly, we could not identify those individuals as medical doctors who worked in managerial positions in the health sector for the whole observation period, because they did not have a “medical doctor” occupation code at any time, so we could not determine whether they were medical doctors.

With the help of the detailed information on labour market status and other data concerning the individuals, five status groups could be distinguished: (1) those working as a physician or dentist in Hungary, (2) out-migrated, (3) exited the profession (attrition), (4) exited employment (related to inactivity, unemployment), or (5) died. We used the following scheme to classify the observations in relation to these groups.

1. Working as medical doctor
  To this group, we classified those individuals who had a “medical doctor, general practitioner”, “medical doctor, specialist doctor” or “medical doctor, dentist” occupation code in the Hungarian classification of occupations code system in the given month. Also, individuals whose professional code was missing in the observed month, but who had any medical doctor professional code in the previous month and whose employer was the same as in the last month were also classified as working doctors. Those individuals whose professional code was missing, but there was at least one month prior to the observed month when the individual had medical doctor occupational code, and whose employer belonged to the health sector were also classified as the working doctors, as well as those persons who were working in the area of health and who had a medical doctor occupational code previously, and whose professional code changed to health service manager codes.
2. Out-migrated
  Those Hungarian citizens who sign on abroad have an obligation to notify the authorities that they have left the country, but many who out-migrate omit this duty. First, we classified to the group of ‘out-migrated’ those who reported their move abroad. We also wanted to identify those who have not notified out-migration. So, in addition, in the out-migrated group were placed all individuals who for at least four successive months were neither registered as employed in the database of the Pensions Directorate, nor were signed in the database of the Health Insurance Fund as being in receipt of inpatient care sickness benefit, and who during that period had received neither any other kind of benefits (unemployment assistance, childcare pension, old age pension or other kind of pensions) nor were they registered as studying in full time education, or died during this period. In other words, the classification covered those individuals who ‘disappeared’ from the system. The other possible reason for the disappearance is that a person who becomes unregistered unemployed is practically non-existent among medical doctors in Hungary. So, it is very likely that using the presented method we were able to identify unnotified out-migrated quite well. Further restrictions were placed on the process of determining the unnotified out-migrated. Only those medical doctors were signed as out-migrated who had worked as a physician or dentist in at least three successive months before the ‘disappearance’. Also, we did not sign those medical doctors as out-migrated whose ‘disappearance’ lasted exactly from the beginning of January until the end of December in a given year and who had worked in the same workplace in the months preceding the disappearance as after the return. We assumed that the employer failed to report the individual for the given year to the Pensions Directorate in these cases. At the same time in the case of some of the omitted observations, the individuals, in fact, might enter employment abroad on a yearly fixed-term contract. Due to these restrictions, we give a lower bound estimate on the out-migration. As a consequence of the procedure of identifying the out-migrated as described above, in the first and last three months of the observation period, the number of out-migrated is likely to be an underestimate, as only the notified out-migrated could be identified in these months.
3. Exited the profession (attrition)
  Those observations were classified in this way who did not have a ‘medical doctor’ occupation code in the observed month and who had not worked in the health sector or did not meet other criteria qualifying them as ‘working as a doctor’.
4. Exited employment
  In this group were placed the unemployed (very few cases, unemployment is practically non-existent among Hungarian medical doctors), those who were in full-time education, who were on a child-care allowance disability pension or retired.
5. Died
  We had direct observations for death in our database (in the database of the Health Insurance Found).

## Methods

We analysed the out-migration decisions of doctors with the help of time-to-event analysis. We used competing risk models [28]. Doctors may leave the domestic health workforce for different reasons: out-migration, a movement to non-health sector employment, retirement, child-care leave, etc. A competing risk is defined as an event whose occurrence precludes or alters the probability of occurrence of the main event under examination. In our case, the individual either migrates, or goes on to a job outside the health sector, becomes inactive or unemployed or dies. Competing risk models define separate hazard functions for each event $h_{k} \left( t \right)$. A cause-specific hazard $h_{k} \left( t \right)$ is the immediate risk of failure from a particular cause (*k*) given that failure (from any cause) has not happened previously

$$h_{k} \left( t \right) = \mathop {\lim }\limits_{\delta \to 0} \frac{{P(t \le T < t + \delta , {\text{event}} = k|T > t)}}{\delta },$$

where *T* is the time to failure from any event. The total hazard is the sum of the sub-hazards:

$$h\left( t \right) = \mathop \sum \limits_{k = 1}^{K} h_{k} (t ),$$

where *K* is the number of competing events.

We used the Fine & Gray model, which is a modified Cox proportional hazard model that allows the presence of competing risks. The Fine & Gray model makes possible the direct modelling of the effects of covariates on the cumulative incidence function via a semiparametric approach. Based on the relationship between the hazard and survival functions, the model defines subdistribution hazards instead of cause-specific hazards. The fundamental difference between a cause-specific hazard and the subdistribution hazard is the risk set. The subdistribution hazard, *h*_*k*sub_ (*t*), is the immediate risk of leaving the profession on account of a particular cause, *k*, given that the subject has not left the job before as a result of cause *k*.

$$h_{{k{\text{sub}}}} \left( t \right) = \mathop {\lim }\limits_{\delta \to 0} \frac{{P\left( {t \le T < t + \delta , {\text{event}} = k |T > t} \right)\,\,{\text{or }}\left( {T \le t \& K \ne k} \right)}}{\delta }$$

As in the Cox model, the Fine & Gray model for failure is also a relative risk model that decomposes into a baseline hazard and the regression effects of covariates:

$$h\left( {t|X} \right)_{\text{sub}}\,\, = h_{0} \left( t \right)_{\text{sub}} \exp \left( {\beta_{\text{sub}}^{T} x} \right),$$

where $h_{0} (t)_{\text{sub}}$ is the baseline subdistribution hazard and $\beta_{{{\text{sub }} }}$ is the vector of covariate effects (log subhazard ratios). Similarly to the Cox model, the Fine & Gray model makes no assumptions about the baseline hazard function and assumes proportional hazards.

Based on the predictions of the competing risk model the cause-specific cumulative incidence function, ${\text{CIF}}_{k } \left( t \right)$ gives the proportion of doctors at time *t* who have left the profession for a cause k, accounting for the fact that the job can also be left for other causes:

$${\text{CIF}}_{k } \left( t \right) = P(T \le t,{\text{event}} = k).$$

The total cumulative incidence function is the sum of cause-specific incidence functions:

$${\text{CIF}}\left( t \right) = \mathop \sum \limits_{k = 1}^{K} {\text{CIF}}_{k} (t)$$

In the analysis we distinguished four competing risk events: (1) out-migration, (2) exits from the profession (attrition), (3) exits from employment (related to inactivity and unemployment), or (4) death.

We conducted the analysis for the whole sample and also for subsamples of four age groups. The independent variables were gender, age (in the models that used the entire sample), dummy variables indicating whether the individual was a general practitioner or a specialist doctor versus a dentist, and dummy variables accounting for the region of residence. A further variable verified the relative labour income of the individual that is the average labour income of the person in the preceding three months as a ratio of the average national labour income during the same period (calculated from the source sample). To determine whether the example of peers strengthened out-migration decisions, we included a variable indicating whether any medical doctors had out-migrated during the preceding three months from the same workplace where the individual had been working before migrating. This covariate may also reflect the effect of the unobserved differences in working conditions among different workplaces. We also inserted two dummy variables into the models indicating the month of Hungary’s EU accession (05.2004) and another indicating the month (05.2011) when the transitional period of restrictions on the free movement of labour from EU-8^6^ countries to Austria and Germany ended.

In order to analyse how the probability of return migration of the out-migrated medical doctors may have changed, a binary choice Cox proportional hazard model (returning to the profession in Hungary or not) was used. In the Cox model, the risk given covariates are the product of the baseline hazard and a relative risk. In this model, the following were included as independent variables: gender, age group dummies and dummy variables indicating whether the individual was a general practitioner or a specialist doctor as opposed to a dentist.

## Results

### Where have all the doctors gone?

**Fig. 1:**
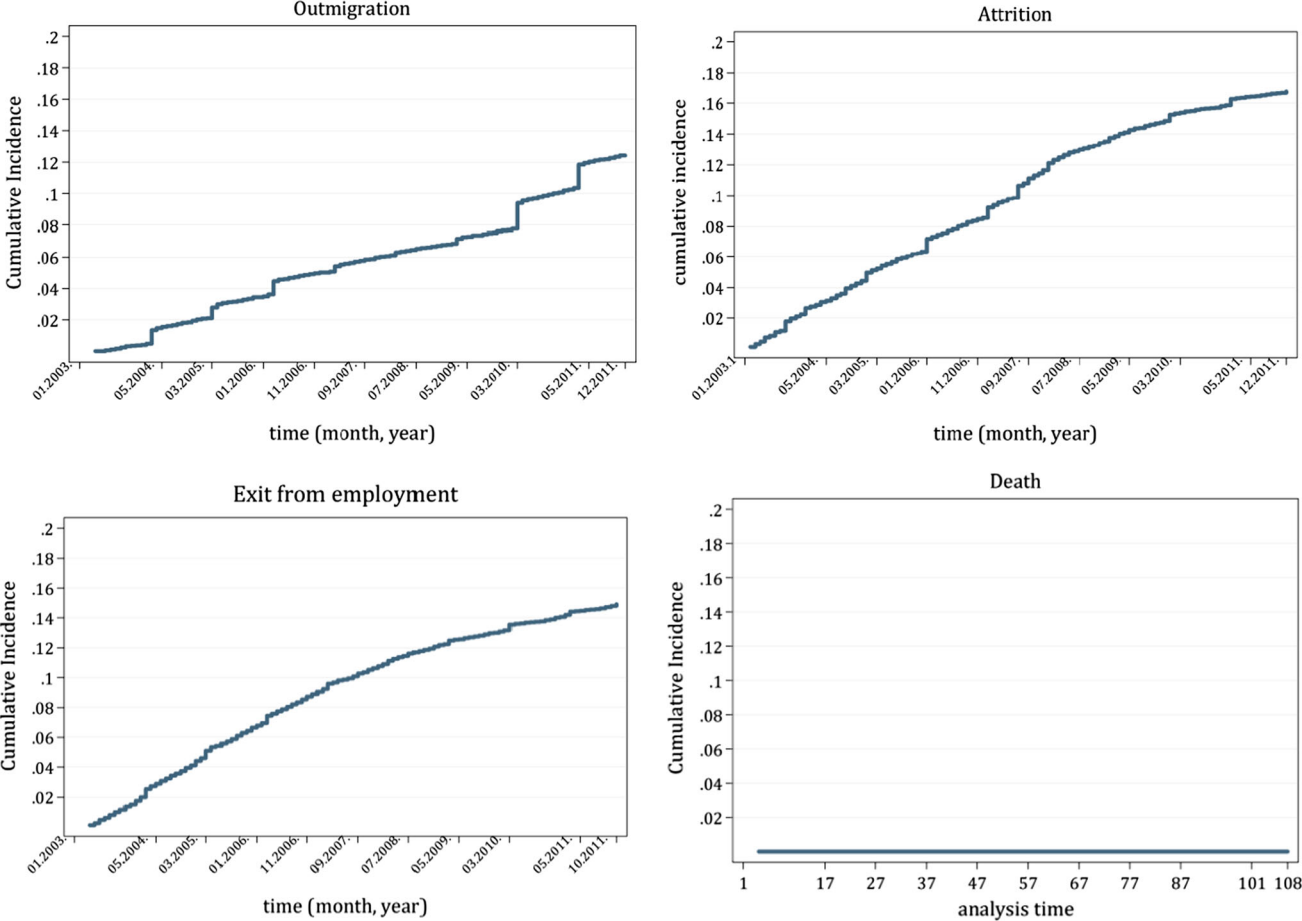
Cumulative incidence functions: out-migration/attrition/exit from employment/death

First, we present the cumulative incidence rates of the four competing outcomes based on the models for the whole sample (Fig. 1). These are the cumulative probabilities of exit via each of the competing risk events: out-migration, attrition, exiting employment, and death. The probability of leaving the medical workforce due to death is negligible, so we will not present detailed results for this outcome in the following. Between January 2003 and December 2011, 12% of the practising medical doctors left the country, 17% left the domestic health workforce and went to a job outside the health sector in Hungary, and about 14% became inactive (retired, were placed on disability pension or child-care pension or became unemployed). A part of this group, those who were in receipt of child-care allowance returned to healthcare some time later, the average duration of maternity leave^7^ being 13 months in the sample. The cumulative incidence functions indicate that attrition contributed to doctor shortages even more than out-migration in the observation period.

Table 1 summarises the results of the separate competing risk models for out-migration. The first column of the table shows the results for the whole sample; columns 2–5 indicate the results for the age-group-specific subsamples. A sub-hazard rate greater than one implies a higher probability of out-migration compared to the reference category, while a rate of less than one indicates a lower probability. For instance, in the model for the whole sample (column 1), the sub-hazard rate for gender is 1.22, indicating that the probability of out-migration of men is 22% higher than for women. Similarly, a 1% increase in relative labour income decreased the probability of emigration by 6% (the sub-hazard rate is 0.94).

**Table 1:** Competing risk models (subhazard rates) — out-migration (competing risks: attrition/exit from employment/death)

Variable	Subhazard rates				
	The whole sample	<31 years old	31–40 years old	41–50 years old	51–60 years old
Gender (male = 1)	1.22^a^ (0.058)	1.37 (0.164)	1.49^a^ (0.133)	1.21 (0.130)	0.76^b^ (0.070)
Age	0.99^a^ (0.002)	–	–	–	–
General practitioner	1.33^a^ (0.076)	2.17^a^ (0.309)	1.86^a^ (0.182)	1.39 (0.225)	0.82 (0.123)
Specialist doctor	1.04 (0.060)	0.98 (0.245)	1.62^a^ (0.163)	1.21 (0.141)	0.83 (0.079)
Relative labour income	0.93^a^ (0.027)	0.46^a^ (0.074)	0.68^a^ (0.062)	0.90 (0.062)	1.04 (0.036)
Peer effect	1.15 (0.063)	0.76 (0.098)	1.37^a^ (0.130)	1.34 (0.178)	1.00 (0.117)
Region: Central Transdanubia	1.41^a^ (0.152)	1.45 (0.572)	1.53 (0.279)	1.30 (0.299)	1.43 (0.267)
Region: Western Transdanubia	0.85 (0.116)	0.99 (0.449)	0.63 (0.171)	1.01 (0.265)	1.02 (0.227)
Region: Southern Transdanubia	0.52^b^ (0.120)	0.31 (0.316)	0.67 (0.220)	0 (0)	0.78 (0.294)
Region: Northern Hungary	0.75 (0.094)	0.40 (0.235)	0.90 (0.179)	0.52 (0.161)	0.86 (0.188)
Region: Northern Great Plain	1.08 (0.109)	2.33^a^ (0.581)	0.82 (0.151)	1.08 (0.223)	0.85 (0.178)
Region: Southern Great Plain	0.89 (0.136)	1.42 (0.578)	0.67 (0.193)	1.17 (0.332)	0.86 (0.248)
EU accession (May 2004)	0.51 (0.159)	0.00 (0)	0.27 (0.169)	0.32 (0.199)	0.34 (0.161)
Lifting of temporary restrictions (May 2011)	5.75^a^ (1.029)	7.65^a^ (3.069)	6.72^a^ (2.330)	4.04^b^ (1.848)	0.00^a^ (0)

Standard errors in parentheses

Reference category: female; dentist; Central Hungary; month other than 05.2004; month other than 05.2013

^a^Significant at the 1% level

^b^Significant at the 5% level

For the whole sample, the results show that EU accession did not change the probability of the out-migration of Hungarian medical doctors, but the lifting of the Austrian and German restrictions on the free movement of labour from EU-8 countries significantly affected the probability of Hungarian doctors’ emigration. There might be different explanations for this increase: historical and cultural ties between Austria, Germany and Hungary may have had an effect, as well as the fact of geographical closeness, but this question needs further research. For the whole sample, there was a more than fivefold increase in the probability of out-migration in May 2011. The results of the age-specific subsamples show that EU accession affected the probability of out-migration for none of the age groups, but the effect of the lifting the Austrian and German restrictions was significant for all age groups. The increase in the probability of out-migration was larger for the young. There was a more than a sevenfold increase for medical doctors under 31, and more than sixfold for physicians and dentists in the 31–40 age bracket. Nevertheless, the probability of out-migration of physicians and dentists even in the 41–50 range increased fourfold. The effect was zero and the zero effect was significant. In the 31–40 age group, there was a significantly higher probability of men out-migrating than for women. Among the 51- to 60-year-olds, the probability of out-migration was greater for women than men doctors. In the other age groups, there were no significant differences in the probability of out-migration by gender. Relative labour income exercised a significant effect on the out-migration decisions of young medical doctors, namely those under 31 years old, as was also the case for the 31- to 40-year-olds. In these age groups, the lower the relative income of the medical doctor, the larger is the probability of out-migration. For the 31- to 40-year-olds, peer pressure also plays a significant role. Medical doctors move abroad with greater probability if any medical doctors from the same workplace had out-migrated during the preceding 3 months. This might reflect the effects of “peer pressure” or chain migration, but it could also be the consequence of deteriorating working conditions in the given workplace resulting from understaffing on account of out-migration. This question also needs further research. General practitioners also move elsewhere with higher probability than the reference group (dentists). Regional differences are not typical in the probability of out-migration.

**Fig. 2:**
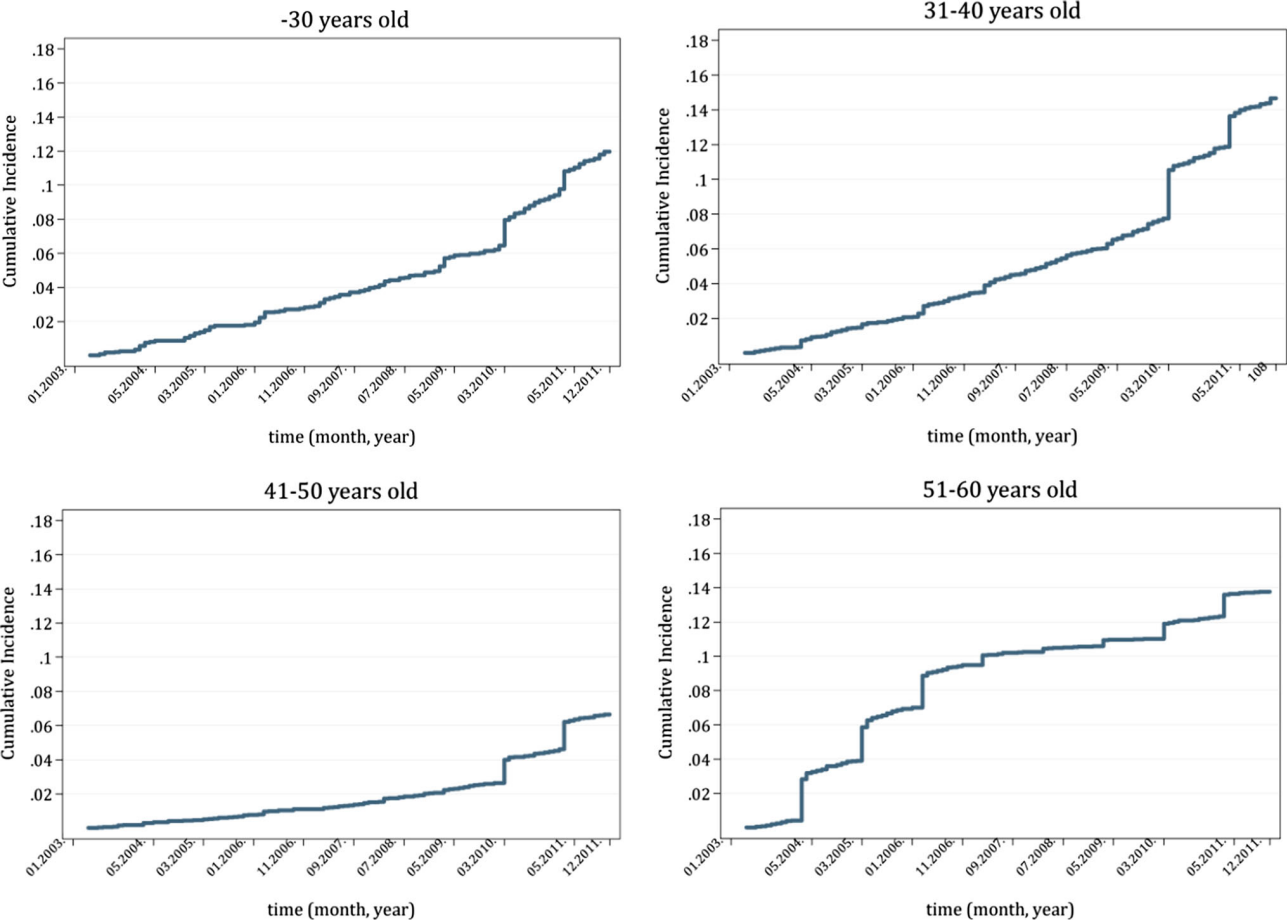
Cumulative incidence functions — out-migration of medical doctors by age groups

Changes in the dynamics of the probability of out-migration in the observed period can be traced with the help of the cumulative incidence functions. Figure 2 shows the cumulative incidence functions of out-migration for the different age groups as predicted by the competing risk models.

Between 2003 and 2012 more than 14% of 31- to 40-year-old Hungarian medical doctors and dentists left Hungary, 12% of those younger than 31, and 14% even of those in the 51–60 age group. The group of doctors aged 41–50 shows the lowest rates. Immediately upon EU accession, doctors of the oldest age group, 51–60 years, left the country at the fastest pace, probably because they could take advantage of their previous professional contacts in finding suitable jobs. After that, until March 2010, the outflow of the 51–60 age group stopped. In the case of the other age groups, there was a steady outflow until March 2010: by that date, 8% of the 31- to 40-year-olds, 6% of those younger than 31, and only 3% of those in the 41–50 age group went abroad. It was not, however, only the end of the transitional period (May 2011) of restrictions on the free movement of labour from EU-8 countries to Austria and Germany that increased the probability of out-migration of medical doctors of all age groups: another turning point may be observed in March 2010. There are several possible explanations for this sudden change. It might be the effect of the economic crisis or the forecast results of the upcoming general elections in Hungary in April 2010 that might have contributed to that change. In fact, in March 2010, the rise in the outflow was higher than the increase in May 2011. After March 2010, the probability of out-migration rose steeply not only for the younger medical doctors but also for the 41- to 50-year-olds as well. The rise of out-migration after March 2010 may highlight that not only the pull factors play a decisive role in out-migration decisions, but the push factors might be equally important in these decisions. The results of the competing risk models for the attrition of medical doctors in Hungary during the same period confirm this explanation.

**Fig. 3:**
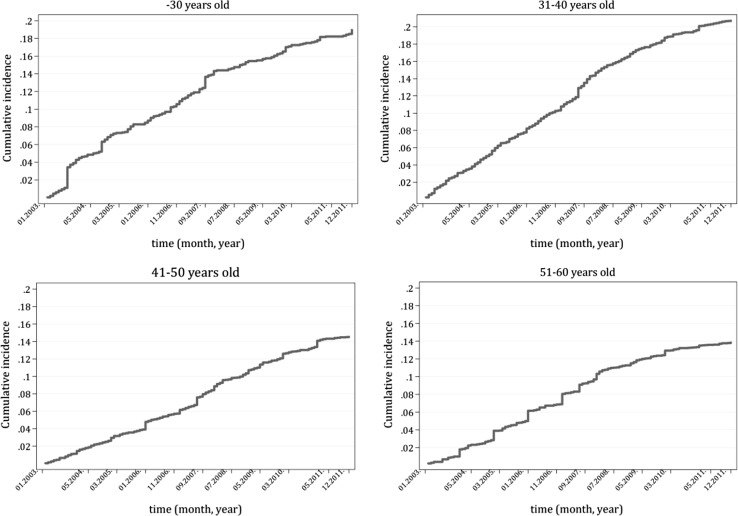
Cumulative incidence functions — attrition of medical doctors by age groups

Figure 3 presents the cumulative incidence functions of attrition for the different age groups. In the examined period, a larger fraction of young doctors left the health sector than that of the older age groups. Eighteen per cent of physicians and dentists younger than 31 years old and 20% of medical doctors aged 31–40 left their profession and took up another job in Hungary. During the same period, 14% of both the 41–50 and 51- to 60-year-old doctors also left the job. In all age groups, a larger proportion of doctors found a job outside the health sector in Hungary than that which out-migrated.

Table 2 summarizes the results of the separate competing risk models of attrition. There are no gender differences in the probability of attrition in the whole sample, and for the different age groups, but for the 31- to 40-year-olds. In this age group, women go to a job outside the health sector with a significantly larger probability than do men. The probability of general practitioners and specialist doctors leaving the profession is smaller than in the case of dentists. Relative labour income also has a significant effect on the probability of attrition for the whole sample as well as for all of the age groups. The larger the relative income of a medical doctor, the larger the probability is that she/he will go to a job outside the health sector even if we control for age, gender, and the other explanatory variables. This larger income is probably in large measure the result of more overtime and night duty work. Medical doctors who choose to work additional night and weekend shifts to increase their salary or who are better paid for other reasons stand a greater probability of leaving the profession. It seems that demanding working conditions play a decisive role in attrition decisions. For older doctors there might also be other considerations for leaving the profession. Official pay in the Hungarian health sector is low, and even declined during the crisis. Medical doctors earn significantly less than graduates with a similar education and of similar age employed in industry. Although actual labour income including “thank-you money” is necessarily higher than customary pay, this extra pay varies widely with age, rank and field of practice [25]. Younger doctors usually get small amounts of “thank-you money”, while older doctors get much more, but they include only a part of the real amount of such payments in their tax statements. In Hungary, there is a uniform, mandatory, earnings-related public pension system and the hidden income is naturally not counted in the income of the individual when the earnings-related pension is calculated. The desire to increase their legal income before retirement may harden the attrition decisions of older doctors.

**Table 2:** Competing risk models (subhazard rates) — attrition (competing risks: out-migration/exit from employment/death)

Variable	Subhazard rates				
	The whole sample	<30 years old	31–40 years old	41–50 years old	51–60 years old
Gender (male = 1)	0.89 (0.036)	0.77 (0.083)	0.67^a^ (0.049)	0.98 (0.077)	0.96 (0.081)
Age	0.99^a^ (0.002)	–			
General practitioner	0.64^a^ (0.034)	0.59^a^ (0.061)	0.73^a^ (0.065)	0.53^a^ (0.071)	0.41^a^ (0.071)
Specialist doctor	0.62^a^ (0.027)	0.61 (0.120)	0.71^a^ (0.057)	0.48^a^ (0.038)	0.64^a^ (0.055)
Relative labour income	1.12^a^ (0.014)	1.56^a^ (0.085)	1.18^a^ (0.030)	1.15^a^ (0.027)	1.10^a^ (0.016)
Peer effect	0.97 (0.048)	1.35^b^ (0.143)	0.70^a^ (0.068)	0.87 (0.090)	0.97 (0.111)
Region: Central Transdanubia	0.97 (0.100)	0.83 (0.254)	0.96 (0.169)	0.80 (0.162)	1.10 (0.220)
Region: Western Transdanubia	1.07 (0.110)	0.91 (0.286)	1.13 (0.202)	0.82 (0.171)	1.50 (0.266)
Region: Southern Transdanubia	0.69 (0.114)	0.45 (0.261)	0.85 (0.225)	0.73 (0.202)	0.54 (0.213)
Region: Northern Hungary	0.75 (0.079)	0.95 (0.242)	0.68 (0.134)	0.59 (0.122)	1.08 (0.203)
Region: Northern Great Plain	0.94 (0.085)	0.73 (0.212)	0.94 (0.150)	0.95 (0.155)	1.06 (0.192)
Region: Southern Great Plain	0.90 (0.112)	1.09 (0.347)	1.10 (0.215)	0.64 (0.161)	1.10 (0.264)
EU accession (May 2004)	0.44 (0.114)	0.82 (0.335)	0.43 (0.156)	0.48 (0.268)	0 (0)
Lifting of temporary restrictions (May 2011)	4.85 (0.752)	0 (0)	6.47^b^ (2.668)	1.06 (0.090)	9.63 (6.774)

Standard errors in parentheses

Reference category: female; dentist; Central Hungary; month other than 05.2004; month other than 05.2013

^a^Significant at the 1% level

^b^Significant at the 5% level

To obtain a fuller picture of the determinants of attrition it is worth summarizing labour income gains of former doctors and looking at where former medical doctors work after having left the medical workforce. Table 3 shows the average monthly labour income gains of former doctors in the 1st month after they left the profession and the income benefit as a percent of current physicians’ employment income (in December 2011 prices). The revenue gains resulting from job changes are rather high. On average, those medical doctors who left the profession enjoyed a 40% higher labour income than before the change in job. The employment income gain was greater for the older physicians or dentists. On average, the pay increase attributable to attrition was about 52% for the 50- to 60-year-olds and about 30% for the youngest doctors.

**Table 3:** Average monthly labour income gain of Hungarian medical doctors in the 1st month after attrition

Age group	Average monthly labour income gain thousands HUF (in 2011 prices)	Average labour income gains as a % of average labour income of medical doctors
<30 years old	63,553	30.4
31–40 years old	68,137	26.9
41–50 years old	109,136	38.8
51–60 years old	158,226	51.8
Total	109,583	39.9

Table 4 summarizes the distribution of former medical doctors who are working outside the health sector by sector of employment, and Table 5 by occupation groups after they leave the health sector. A quarter of former doctors find a job in education and research. These jobs are very likely to be connected to their physician qualification, as are, more or less, the jobs in sectors related to pharmaceutical commerce, where 35% of former doctors work. About 40% of former doctors, however, find jobs outside the health sector. Very similar patterns can be observed in the distribution by occupations of former medical doctors. About 42% of them go to another job that is not connected with their qualification (Table 5).

**Table 4:** Distribution of former medical doctors by sector of employment after attrition (percent)

Sector	Percent
Sectors related to pharmaceutical commerce	35.0
Education and research	25.0
Other sectors	40.0
Total	100

**Table 5:** Distribution of former medical doctors by occupation after attrition (percent)

Occupation group	Percent
Managerial jobs outside the health sector	18.5
Occupations related to pharmaceuticals	20.7
Occupations related to pharmaceutical commerce	18.5
Other occupations outside the health sector	42.3
Total	100

Besides out-migration and attrition, leaving employment also contributes — at least temporarily — to the growing shortage of medical doctors in Hungary. The majority of medical doctors who became inactive during the examined period did so not primarily as a result of retirement, but were chiefly woman medical doctors who were on maternity leave for a while (see Appendix 2 Table 8; Fig. 5). Although women doctors return to practice on average about a year later, the growing feminization of the profession may enhance the effect of these absences on medical doctor shortages.

### Return migration

**Fig. 4:**
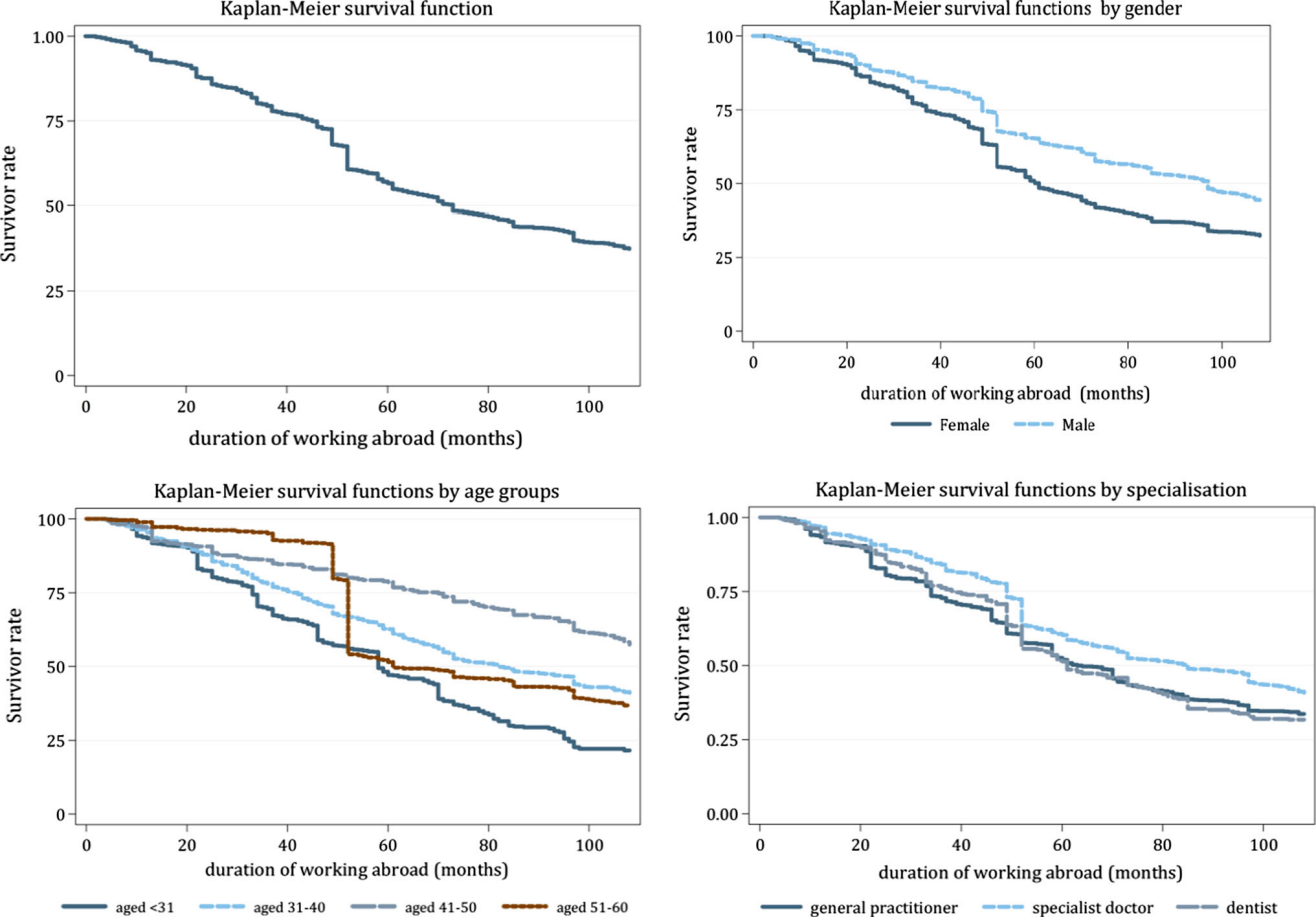
Return migration of out-migrated medical doctors — Kaplan–Meier survival functions

**Fig. 5:**
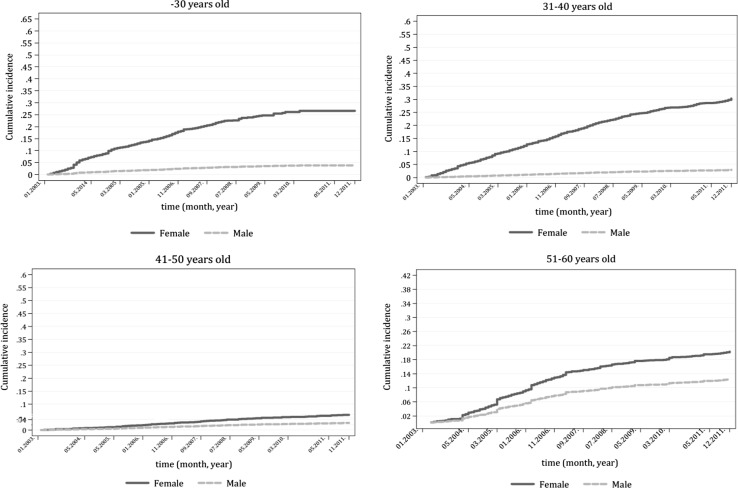
Cumulative incidence functions — exit from employment of medical doctors by age groups and gender

Some of the medical doctors who had left the country during the observation period returned some time later. Figure 4 shows the empirical Kaplan–Meier survival functions which represent the survival times abroad, that is, the proportion of out-migrated medical doctors who are still working abroad a certain amount of time after out-migration. The figure shows the Kaplan–Meier curves for the entire sample and also the curves by gender, by age group and by the specialisation of the medical doctors. Among medical doctors who left the country between January 2001 and December 2011, the average duration of working abroad was 54 months. In the case of the youngest doctors, those younger than 31 years old, the duration of their work abroad was the shortest, on average 42 months. For the 31- to 40-year-olds and the 41–50 age-group the average duration was 59 months, and for the 51–60 bracket, 53 months. A possible explanation of why the youngest doctors spent the shortest time in their foreign jobs and why the greatest proportion of them returned to the Hungarian health sector is that the majority of them left the country for educational opportunities and then they returned home after finishing their studies. Of the 41- to 50-year-old out-migrated doctors, about 60% were still working abroad after 108 months, and so were about 45% of the 31- to 40-year-olds and 40% of the 51- to 60-year old doctors. Those doctors might be considered permanently out-migrated who are not likely to return to the Hungarian health sector. The figure for the whole sample and the gender- and specialisation-specific curves show that the unconditional exit rate from the foreign job was quite steady between the 1st and 48th months, then between the 49th and 50th month there was a sudden increase in the exit rate, and thereafter the exit rate slowed down. The exit rates by gender show that the difference in the exit rates was small between the 1st and 50th months. At that point and beyond, a larger proportion of women left their foreign jobs than did men. The curves by age groups provide an explanation for this. It was the oldest age group, the 51- to 60-year-olds whose exit rate accelerated significantly around the 50th month. It is very likely that a large proportion of medical doctors attaining retirement age return home and work for a while in the Hungarian health sector.

Table 6 presents the results of the Cox proportional hazard model of return migration. Being male decreases the probability of return migration by 20%, as compared to that for women. The older the out-migrated doctor is, the smaller is the probability that he/she will return. The hazard of return migration is 97% smaller for general practitioners and 99% smaller for specialist doctors than for dentists. There is a 46% higher probability of the youngest doctors returning home than their counterparts in the other age groups, and there is no significant difference in the probability of leaving the foreign job comparing 31–40, 40–50 and 51- to 60-year-old medical doctors.

**Table 6:** Return migration of out-migrated medical doctors — Cox proportional hazard model

Variable	Hazard rate
Male	0.82^a^ (0.041)
<31 years old	1.46^a^ (0.104)
31–40 years old	1.14 (0.082)
41–60 years old	1.06 (0.083)
General practitioner	0.04^a^ (0.008)
Specialist doctor	0.02^a^ (0.005)
Number of observations: 187,195	
Number of subjects: 2749	
No. of failures: 1713	
Time at risk: 187,195	

Robust standard errors in parentheses

Reference group: female; dentist, 41–50 years old

^a^Significant at the 1% level

A number of the returning medical doctors may out-migrate again at a later date. In our sample, 90% of the out-migrated doctors took a job abroad once during the observed 108 months and only the remaining 10% out-migrated twice or more during this period. Our results show that about half of the out-migrated doctors are permanent migrants.

## Conclusions

The enlargement of the EU amplified concerns in the new member states that the increased job-opportunities for doctors in other countries within the EU offering attractive working conditions would lead to increasing shortages of doctors in the new member states, and thus have an adverse effect on the health systems of these countries. Nevertheless, discussion of the effect of EU enlargement on the labour migration of medical doctors is severely hindered by the lack of comprehensive data which would allow a more accurate monitoring of health labour migration.

The main contribution of this study to existing research is that we were able to exploit a large, linked, individual-level, administrative panel dataset in order to analyse changes in the dynamics of migration of Hungarian medical doctors between 2003 and 2011 on a monthly basis. Panel data have a greater capacity for capturing the complexity of migration processes than a simple cross-section database. The most significant advantage of panel data is the possibility of being able to track individuals over time. We were able to observe how much time the status (e.g. actually working as a doctor) of a particular medical doctor lasted, in which period they changed their labour market status (including migration), and also observe the return migration of medical doctors. Data on the return migration of medical doctors are even less complete, a fact further hampered by the dynamic and complex nature of migration itself. An additional advantage of our database was the length of the observation period, 9 years, that is, 108 months. The period included months before and after EU accession, and months before and after Austria and Germany lifted restrictions on the free movement of labour from new member states. As our sample was based on administrative data, we did not have to face the potential attrition bias which is a common problem with panel data, that is, cases when some observations from the baseline period are missing in the follow-up periods, and we were also able to use this characteristic of our sample to identify migration. Nevertheless, administrative data has not only benefits, but limitations too. Administrative data systems are not designed for statistical purposes or scientific research, and they collect information only for their specific purposes, so we had only a limited number of control variables. For instance, we had no data on the specialisations of specialist doctors, or the family status of medical doctors, which also might have had an effect on migration decisions. Our data did not capture those cases of the mobility of medical doctors when they take up short-term contracts and participate in the systems of two countries, i.e. those who work abroad, but do so on a part-time basis while also being employed in Hungary. Our results apply only to those medical doctors who had left the Hungarian health system and worked abroad.

The results of this study show that EU accession did not affect the out-migration of Hungarian physicians and dentists, but after Austria and Germany lifted the temporary restrictions towards workers from the new EU member states in May 2011, the migration of medical doctors increased considerably. We also found that push factors have as great a role in the out-migration decisions of Hungarian physicians and dentists as pull factors. Medical healthcare shortages are not only due to high outward migration but to other problems in the Hungarian health system: attrition and feminization. Outward migration plays an important and growing role in the shortages. The scale of return migration is also considerable. The Hungarian health system can gain from out-migration, however, only if the returning medical doctors can put their increased skills and knowledge to use. If attrition and out-migration continue at the same pace as hitherto, and the facilities remain understaffed, a decrease in the quality of care will be difficult to stop.

Although our research used a specific Hungarian data set, it seems likely that some of these findings would be similar in other EU-10 countries. The results of several case studies and summaries of PROMeTHEUS and MoHProf projects show that in most of the EU-10 countries the migration of health professionals had already started well before those countries’ EU accessions [12, 21] due to the political transition in the 1990s, and that post-accession outflows from the EU10 have been lower than expected. It seems that in other EU-10 countries, too, shortages of medical doctors are due not only to high outward migration, but also to other problems in the health system, such as attrition. Nevertheless, we cannot assess how the dynamics of the migration of medical doctors has changed in these countries because of the lack of detailed and comparable data on outflows and inflows. There are attempts in some EU countries to collect detailed survey data regularly on the emigration of medical doctors and return migration, e.g. Iceland [29]. To create a comparable, detailed register of such migration in EU countries, or the regular monitoring of this migration in the EU with large-scale surveys might help us better understand the dynamic nature of migration and assist in the adoption of adequate policy measures.

## Appendix 1

See Table 7.

**Table 7:** Summary statistics

Variable	The whole sample		<30 years old		31–40 years old		41–50 years old		51–60 years old	
	Mean	Standard deviation	Mean	Standard deviation	Mean	Standard deviation	Mean	Standard deviation	Mean	Standard deviation
Male	0.45	0.497	0.39	0.487	0.45	0.497	0.43	0.494	0.45	0.497
Age	43.38	13.450	–	–	–	–	–	–	–	–
General practitioner	0.17	0.373	0.30	0.458	0.20	0.403	0.11	0.312	0.11	0.309
Specialist doctor	0.33	0.471	0.05	0.211	0.31	0.463	0.51	0.500	0.47	0.499
Dentist	0.07	0.247	0.05	0.227	0.09	0.284	0.07	0.252	0.06	0.241
Relative labour income	1.66	1.255	1.34	0.801	1.63	1.214	1.80	1.346	1.87	1.311
Peer effect	0.24	0.429	0.42	0.494	0.25	0.434	0.20	0.399	0.2	0.403
Region: Central Hungary	0.23	0.422	0.10	0.306	0.26	0.441	0.27	0.443	0.3	0.46
Region: Central Transdanubia	0.03	0.181	0.02	0.126	0.04	0.203	0.04	0.198	0.04	0.201
Region: Western Transdanubia	0.03	0.175	0.01	0.116	0.04	0.184	0.04	0.197	0.04	0.202
Region: Southern Transdanubia	0.02	0.129	0.01	0.094	0.02	0.142	0.02	0.148	0.02	0.131
Region: Northern Hungary	0.04	0.196	0.02	0.148	0.04	0.205	0.05	0.226	0.05	0.216
Region: Northern Great Plain	0.05	0.213	0.02	0.149	0.06	0.239	0.06	0.240	0.05	0.224
Region: Southern Great Plain	0.02	0.152	0.01	0.110	0.03	0.170	0.03	0.174	0.03	0.163
EU accession (May 2004)	0.01	0.097	0.01	0.112	0.01	0.096	0.01	0.098	0.01	0.09
Lifting of temporary restrictions (May 2011)	0.01	0.097	0.01	0.071	0.01	0.098	0.01	0.096	0.01	0.102

## Appendix 2

See Fig. 5 and Table 8.

**Table 8:** Competing risk models (subhazard rates) — exit from employment (competing risks: out-migration/attrition/death)

Variable	Subhazard rates				
	The whole sample	<30 years old	31–40 years old	41–50 years old	51–60 years old
Gender (male = 1)	0.36^a^ (0.018)	0.118^a^ (0.020)	0.08^a^ (0.010)	0.41^a^ (0.072)	0.59^a^ (0.053)
Age	1.00 (0.003)	–	–	–	–
General practitioner	1.62^a^ (0.086)	1.835^a^ (0.222)	1.96^a^ (0.173)	2.79 ^a^ (0.545)	0.95 (0.129)
Specialist doctor	0.98 (0.055)	1.565 (0.278)	1.69^a^ (0.153)	1.15 (0.196)	0.92 (0.086)
Relative labour income	0.74^a^ (0.047)	0.157^a^ (0.021)	0.38^a^ (0.045)	1.16^a^ (0.043)	0.98 (0.081)
Peer effect	1.31^a^ (0.074)	1.199 (0.131)	1.48^a^ (0.127)	1.08 (0.216)	0.72 (0.093)
Region: Central Transdanubia	1.01 (0.105)	1.732 (0.374)	0.88 (0.159)	0.88 (0.336)	0.81 (0.178)
Region: Western Transdanubia	0.91 (0.101)	1.148 (0.269)	1.03 (0.196)	0.40 (0.233)	0.90 (0.200)
Region: Southern Transdanubia	0.69 (0.112)	1.042 (0.274)	0.83 (0.204)	0.21 (0.215)	0.82 (0.297)
Region: Northern Hungary	0.83 (0.084)	1.259 (0.232)	0.97 (0.165)	0.34 (0.173)	1.06 (0.197)
Region: Northern Great Plain	1.02 (0.089)	0.758 (0.161)	1.05 (0.150)	1.04 (0.308)	1.24 (0.211)
Region: Southern Great Plain	1.10 (0.131)	0.805 (0.269)	1.56 (0.295)	1.14 (0.451)	1.10 (0.269)
EU accession (May 2004)	0.74 (0.185)	1.173 (0.278)	0.46 (0.241)	0^a^ (0)	1.78 (1.007)
Lifting of temporary restrictions (May 2011)	7.49^a^ (1.528)	–	4.00^b^ (1.894)	11.92^a^ (5.746)	4.22^a^ (1.486)

Standard errors in parentheses

Reference category: female; dentist; Central Hungary; month other than 05.2004; month other than 05.2013

^a^Significant at the 1% level

^b^Significant at the 5% level

## Footnotes

1 EU-10 countries: Bulgaria, Czech Republic, Estonia, Latvia, Lithuania, Hungary, Poland, Romania, Slovenia, Slovakia.

2 EU-15 countries: Belgium, Denmark, France, Germany, Greece, Ireland, Italy, Luxembourg, the Netherlands, Portugal, Spain, United Kingdom, Austria, Finland, Sweden.

3 Data are not available for all EU10 and EU15 countries. Averages are for those countries for which data are available.

4 MoHProf reviewed the trends in international migration of health workers in 25 countries around the world, with a focus on migration within, to and from the EU [15].

5 PROMeTHEUS, looked at health professional mobility and health systems of 17 countries in Europe [16].

6 EU-8 countries: Czech Republic, Estonia, Latvia, Lithuania, Hungary, Poland, Slovenia and Slovakia.

7 Maternity leave is long in Hungary. New parents are entitled to 3 years of maternity leave during which they cannot be dismissed. Maternity leave qualifies the person taking it to other benefits, including pensions.
